# Downregulation of RhoA/ROCK1/YAP/F-actin causing decreased aortic smooth muscle cell stiffness promotes aortic dissection formation

**DOI:** 10.1093/lifemeta/loae022

**Published:** 2024-06-03

**Authors:** Wei Zhang, Mengxiao Wang, Enci Wang, Wei Lu, Zengxia Li, Yuchong Zhang, Gaofei Hu, Qi Zhang, Wenxin Shan, Yongjun Dang, Zhe Zhao, Lemin Zheng, Weiguo Fu, Lixin Wang

**Affiliations:** Department of Vascular Surgery, Zhongshan Hospital, Fudan University, Shanghai 200032, China; Vascular Surgery Institute, Zhongshan Hospital, Fudan University, Shanghai 200032, China; The Institute of Cardiovascular Sciences and Institute of Systems Biomedicine, School of Basic Medical Sciences, State Key Laboratory of Vascular Homeostasis and Remodeling, NHC Key Laboratory of Cardiovascular Molecular Biology and Regulatory Peptides, Beijing Key Laboratory of Cardiovascular Receptors Research, Health Science Center, Peking University, Beijing 100191, China; Department of Vascular Surgery, Zhongshan Hospital, Fudan University, Shanghai 200032, China; Vascular Surgery Institute, Zhongshan Hospital, Fudan University, Shanghai 200032, China; Department of Hemangioma and Vascular Malformation, Plastic Surgery Hospital, Peking Union Medical College, Beijing 100144, China; Department of Biochemistry and Molecular Biology, School of Basic Medical Sciences, Fudan University, Shanghai 200032, China; Department of Vascular Surgery, Zhongshan Hospital, Fudan University, Shanghai 200032, China; Vascular Surgery Institute, Zhongshan Hospital, Fudan University, Shanghai 200032, China; The Institute of Cardiovascular Sciences and Institute of Systems Biomedicine, School of Basic Medical Sciences, State Key Laboratory of Vascular Homeostasis and Remodeling, NHC Key Laboratory of Cardiovascular Molecular Biology and Regulatory Peptides, Beijing Key Laboratory of Cardiovascular Receptors Research, Health Science Center, Peking University, Beijing 100191, China; The Institute of Cardiovascular Sciences and Institute of Systems Biomedicine, School of Basic Medical Sciences, State Key Laboratory of Vascular Homeostasis and Remodeling, NHC Key Laboratory of Cardiovascular Molecular Biology and Regulatory Peptides, Beijing Key Laboratory of Cardiovascular Receptors Research, Health Science Center, Peking University, Beijing 100191, China; The Institute of Cardiovascular Sciences and Institute of Systems Biomedicine, School of Basic Medical Sciences, State Key Laboratory of Vascular Homeostasis and Remodeling, NHC Key Laboratory of Cardiovascular Molecular Biology and Regulatory Peptides, Beijing Key Laboratory of Cardiovascular Receptors Research, Health Science Center, Peking University, Beijing 100191, China; Department of Biochemistry and Molecular Biology, School of Basic Medical Sciences, Fudan University, Shanghai 200032, China; Department of Pharmacy, Peking University Third Hospital, Beijing 100191, China; The Institute of Cardiovascular Sciences and Institute of Systems Biomedicine, School of Basic Medical Sciences, State Key Laboratory of Vascular Homeostasis and Remodeling, NHC Key Laboratory of Cardiovascular Molecular Biology and Regulatory Peptides, Beijing Key Laboratory of Cardiovascular Receptors Research, Health Science Center, Peking University, Beijing 100191, China; Department of Vascular Surgery, Zhongshan Hospital, Fudan University, Shanghai 200032, China; Vascular Surgery Institute, Zhongshan Hospital, Fudan University, Shanghai 200032, China; Department of Pharmacy, Peking University Third Hospital, Beijing 100191, China; Department of Vascular Surgery, Zhongshan Hospital, Fudan University, Shanghai 200032, China; Vascular Surgery Institute, Zhongshan Hospital, Fudan University, Shanghai 200032, China; Department of Vascular Surgery, Zhongshan Hospital (Xiamen), Fudan University, Xiamen, Fujian 361015, China

## Abstract

**Figure:**
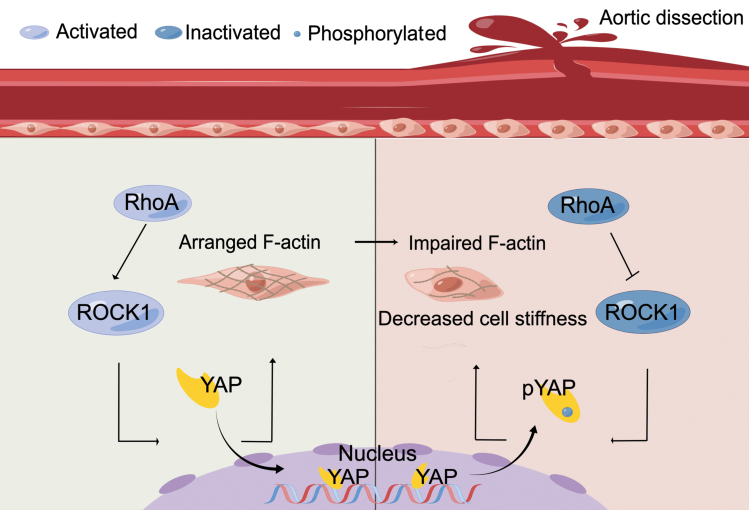
Downregulated RhoA/ROCK1/YAP/F-actin axis leads to decreased AoSMC stiffness and promotes AD formation.

Downregulated RhoA/ROCK1/YAP/F-actin axis leads to decreased AoSMC stiffness and promotes AD formation.

**Dear Editor,**

Aortic dissection (AD) is a fatal disease caused by dysfunction of elastin contractile units, which are composed of extracellular matrix (ECM) and aortic smooth muscle cells (AoSMCs), and eventual disruption of aortic integrity [1]. AoSMCs are the center of the elastin contractile unit, whose phenotype and functionality can be affected by factors that predispose patients to AD, including atherosclerosis, hypertension, smoking, hyperlipidemia, diabetes, and genetics. The intrinsic stiffness of AoSMCs, which is also impacted by these risk factors, plays an important role in regulating vascular tone and ECM remodeling. Approximately 20%−40% of genetic mutations predisposed to AD are associated with AoSMC mechanics [1]. Augmented intrinsic AoSMC stiffness is reported as an adaptive change in aortas with aging and hypertension to prevent aortic diseases [2–5]. However, how intrinsic AoSMC stiffness is altered in AD aortas and its correlation with AD occurrence have not been directly investigated. To explore the alteration and role of intrinsic AoSMC stiffness in AD development, we isolated primary AoSMCs from normal and AD human aortas to compare their intrinsic cell stiffness using atomic force microscopy (AFM).

Intrinsic cell stiffness is related to the cytoskeleton, which is regulated by molecular signaling pathways [6]. RhoA and Rho kinase (ROCK) are known to regulate the cytoskeleton, cellular morphology, and mechanobiological responses. However, their impacts on AoSMC mechanics have not been directly measured except for the influence on the phenotypic transformation of AoSMCs [7, 8]. Yes-associated protein (YAP), a downstream target of RhoA/ROCK1, is also a critical mechanobiological regulator whose activation can orchestrate transcription to modulate F-actin dynamics and cellular mechanics. The RhoA-dependent pathway has been widely studied in several cardiovascular abnormalities associated with atherosclerosis, hypertension, smoking, and diabetes, which are known to play a role in AD. The activities of Rho GTPase (guanine triphosphatase) and YAP are both reported to participate in aortic diseases including AD [4, 9], nevertheless, their correlation with intrinsic AoSMC stiffness remains unclear. To explore the alteration of AoSMC stiffness and its underlying mechanism in AD formation, we compared the expression of RhoA/ROCK1/YAP in primary AoSMCs derived from human normal and AD aortas. The relationship between RhoA/ROCK1/YAP and intrinsic AoSMC stiffness was identified *in vitro* by manipulating the signaling pathway in primary human AoSMCs. Finally, the role of this signaling pathway in AD development was assessed in an animal model.

The expression levels of *RHOA/ROCK1*, *YAP*, and other genes correlated with the RhoA/ROCK1 signaling and YAP signaling pathways were evaluated in the Gene Expression Omnibus (GEO) database of AD and normal aortas. The heatmap showed that the expression of *RHOA*, *ROCK1*, and *YAP* was downregulated in the aortas of patients with AD (Supplementary Fig. S1a). Gene set enrichment analysis (GSEA) verified the downregulation in terms of cyclic adenosine monophosphate (cAMP)-mediated signaling, actin cytoskeleton, ECM organization, and collagen formation in AD aortas (Fig. 1a; Supplementary Fig. S1b). Moreover, we detected decreased expression of YAP in AD aortas (Supplementary Fig. S1c and d). Since AoSMCs are the center of the elastin-contractile unit that maintains aortic integrity, we isolated AoSMCs from normal and AD human aortas to examine the alterations in the ROCK1 and YAP signaling pathways. None of the patients included in our study were diagnosed with hereditary AD. Apart from the history of hypertension (*P* < 0.01), there was no significant difference regarding age, smoking, diabetes mellitus, hemodialysis, history of cardiovascular diseases and relevant medication, or blood fat level between the normal and AD groups (Table 1). The protein levels of RhoA, ROCK1, and YAP were decreased in AD AoSMCs (Fig. 1b). In addition, the phosphorylation of YAP, which indicates the inhibition of YAP activity, was increased in AD AoSMCs when total YAP was used as a reference (Fig. 1b). As an essential effector of biomechanical signaling, YAP can shuttle the nucleus back and forth to mediate gene expression, especially those related to cytoskeleton stabilization that impacts cellular mechanics [10]. Immunofluorescence demonstrated less nuclear localization of activated YAP in AD AoSMCs than in normal AoSMCs (Fig. 1c). Moreover, F-actin filaments were pronouncedly interrupted in AD AoSMCs in contrast to continuous F-actin filaments in normal AoSMCs. A reduced fractal dimension index confirmed impaired cytoskeleton arrangement and decreased structural complexity in AD AoSMCs (Fig. 1c). Given that F-actin is related to the intrinsic cell stiffness of vascular SMCs, we compared the cell stiffness of normal and AD AoSMCs using atomic force microscopy (AFM). Statistical analysis showed that the Young’s modulus of AD AoSMCs was significantly lower than that of normal AoSMCs (Fig. 1d). Therefore, downregulated RhoA/ROCK1 and YAP, impaired F-actin polymerization, and decreased intrinsic cell stiffness were found in AD AoSMCs.

**Table 1.** Comparison of baseline information between the normal and AD groups.

	NA (*n* = 7)	AD (*n* = 13)	*P* value
Age	48.3 ± 5.5	55.4 ± 11.1	0.14
Sex (female)	2	2	0.58
Hypertension	1	12	^**^P < 0.01
Smoking	2	8	0.35
Coronary artery disease	0	3	0.52
Diabetes mellitus	0	2	0.52
Hemodialysis	0	0	
Chronic kidney disease	0	0	
Blood fat level	3	2	0.30
Cardiovascular diseases	0	1	0.99

^**^significance, *P* < 0.01.

NA, normal aorta; AD, aortic dissection.

**Figure 1:**
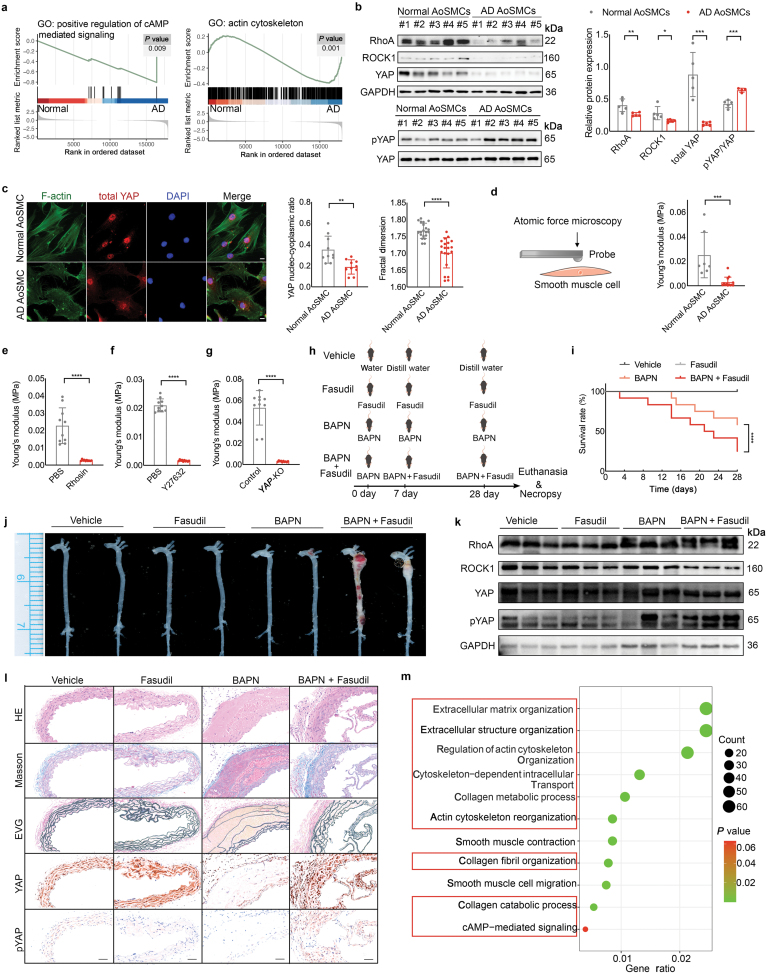
Downregulation of the RhoA/ROCK1/YAP/F-actin axis leads to decreased intrinsic stiffness of AoSMCs, promoting AD formation through increased elastin fragmentation and impaired cell-ECM interactions. (a) GSEA of the AD GEO database (GSE190635) in terms of cAMP-mediated signaling (^**^P < 0.01) and actin cytoskeleton (^**^P < 0.01) in normal and AD aortas. (b) Protein expression levels of RhoA, ROCK1, and YAP in normal and AD AoSMCs. Phosphorylation of YAP in normal and AD AoSMCs was examined when total YAP was used as a reference. Graph: Statistical analysis of relative protein expression. (c) Immunofluorescence of F-actin and YAP in normal and AD AoSMCs. Scale bar = 10 μm. Graphs: Left panel, the nucleocytoplasmic ratio of YAP in normal and AD AoSMCs (^**^P < 0.01); Right panel, the fractal dimension of normal and AD AoSMCs (^****^P < 0.0001). (d) Schematic diagram of cell stiffness measurement using AFM. Graph: Young’s modulus of normal and AD AoSMCs (*n* = 7 in the normal group; *n* = 13 in the AD group. ^***^P < 0.001). (e) Young’s modulus of AoSMCs treated with or without Rhosin (50 μmol/L, 48 h) (^****^P < 0.0001). (f) Young’s modulus of AoSMCs treated with or without Y27632 (10 μmol/L, 48 h) (^****^P < 0.0001). (g) Young’s modulus of control and *YAP*-KO AoSMCs (^****^P < 0.0001). (h) Schematic diagram of AD mouse models induced by BAPN and/or Fasudil. (i) Survival curve analysis of the pharmaceutical AD mouse models. Survival rate: vehicle, 100%; Fasudil, 100%; BAPN, 58%; BAPN + Fasudil, 25%. Statistical analysis: BAPN vs. BAPN + Fasudil, log-rank Mantel-Cox test, ^****^P < 0.0001. (j) Gross photos of harvested aortas. (k) Western blotting analysis of RhoA, ROCK1, YAP, and pYAP in aortas harvested from each group. (l) Aortic cross-sections stained with hematoxylin-eosin (HE), Masson, Verhoeff’s Van Gieson (EVG), anti-YAP, and anti-pYAP immunochemistry. Scale bar = 50 μm. (m) GO analysis of DEGs between the vehicle and BAPN + Fasudil groups.

We have further manipulated the activity of RhoA, ROCK1, and YAP to elucidate the correlations among RhoA/ROCK1, YAP, F-actin, and cell stiffness in normal human AoSMCs. When RhoA activity in normal AoSMCs was inhibited by Rhosin, a RhoA inhibitor, the expression of ROCK1 was subsequently reduced, while the phosphorylation of YAP increased (Supplementary Fig. S2a). Immunofluorescence showed a greater cytoplasmic distribution of YAP following Rhosin treatment, indicating that YAP activity was suppressed (Supplementary Fig. S2b). Additionally, F-actin filaments were poorly arranged and the fractal dimension index was markedly decreased following Rhosin treatment (Supplementary Fig. S2b). Inhibition of RhoA activity also led to a reduction in intrinsic AoSMC stiffness (Fig. 1e). Then, ROCK1 activity was inhibited by Y27632, and phosphorylation of YAP subsequently increased (Supplementary Fig. S2c). Immunofluorescence demonstrated the less nuclear distribution of YAP and severely impaired F-actin arrangement, coupled with a reduced fractal dimension index in AoSMCs treated with Y27632 (Supplementary Fig. S2d). The reduced Young’s modulus of AoSMCs treated with Y27632 was further confirmed by AFM (Fig. 1f). Finally, we depleted *YAP* expression in normal AoSMCs (YAP-KO AoSMCs) and determined that *YAP* deletion did not affect the AoSMC phenotype, based on the expression of alpha-smooth muscle actin (*α*-SMA) (Supplementary Fig. S2e). A disrupted F-actin cytoskeleton with a reduced fractal dimension index was detected in *YAP*-KO AoSMCs (Supplementary Fig. S2f). Moreover, intrinsic AoSMC stiffness was markedly decreased due to YAP ablation (Fig. 1g). Collectively, these results illustrated that downregulation of the RhoA/ROCK1/YAP/F-actin axis leads to decreased intrinsic cell stiffness in AoSMCs.

The impact of inhibiting the RhoA/ROCK1/YAP/F-actin axis on AoSMCs was further explored by RNA sequencing (RNA-seq). Gene Ontology (GO) analysis revealed that differentially expressed genes (DEGs) related to cAMP-mediated signaling, cytoskeleton organization, cell-matrix adhesion, and ECM organization were affected when ROCK1 activity was inhibited by Y27632 in normal AoSMCs (Supplementary Fig. S3a). Kyoto Encyclopedia of Genes and Genomes (KEGG) analysis showed differences in phosphatidylinositol 3-kinase (PI3K)/protein kinase B (PKB/AKT) signaling, focal adhesion, mitogen-activated protein kinase (MAPK), actin cytoskeleton, and ECM-receptor interaction (Supplementary Fig. S3b). Consistently, cAMP, actin cytoskeleton organization, and ECM structural constituents were all downregulated in AoSMCs treated with Y27632 according to GSEA (Supplementary Fig. S3c–e). We also analyzed the downregulated genes in AoSMCs treated with Y27632, and the results demonstrated that the downregulated genes were mainly related to the cell-ECM unit, PI3K, MAPK, focal adhesion, and cAMP signaling pathways (Supplementary Fig. S3f and g). We also found that the expression levels of collagen I and collagen III, two main collagens in the vascular wall, were reduced in AoSMCs treated with Y27632 (Supplementary Fig. S3h). Therefore, inhibition of the RhoA/ROCK1/YAP/F-actin axis led to decreased AoSMC stiffness, thereby compromising the function of the cell-ECM unit, which plays an important role in maintaining aortic integrity.

We further investigated the role of downregulated RhoA/ROCK1/YAP/F-actin with decreased AoSMC stiffness in AD development using an AD mouse model treated with β-aminopropionitrile (BAPN), a traditional medicine for the pharmaceutical AD model. The ROCK1 inhibitor Fasudil was utilized to inhibit the RhoA/ROCK1/YAP/F-actin axis (Fig. 1h). We first identified the impact of Fasudil on normal AoSMCs. Normal human AoSMCs treated with Fasudil showed increased phosphorylation of YAP and impaired F-actin polymerization with a decreased fractal dimension index (Supplementary Fig. S4a and b). Intrinsic AoSMC stiffness was also reduced following Fasudil treatment (Supplementary Fig. S4c). Then we compared the survival rates of mice treated with Fasudil, BAPN, and BAPN + Fasudil. After four weeks of follow-up, no mice died in either the vehicle group or the Fasudil group. Strikingly, the mortality due to AD formation was higher in mice fed BAPN + Fasudil than in mice fed BAPN alone (Fig. 1i), indicating that downregulation of RhoA/ROCK1/YAP/F-actin signaling with decreased AoSMC stiffness can facilitate AD formation. Aortas were isolated to confirm the AD formation, and obvious AD lesions were observed in the BAPN and BAPN + Fasudil groups (Fig. 1j). The aortic diameter in the BAPN and BAPN + Fasudil groups showed a slight increase during the follow-up (Supplementary Fig. S4d). Additionally, the blood pressure and heart rate showed no obvious changes in each group at the beginning and end points of follow-up (Supplementary Fig. S4e and f). Western blotting analysis showed that the levels of RhoA/ROCK1/YAP were downregulated in the aortas derived from Fasudil and BAPN + Faudil groups, confirming the inhibition of ROCK1 by Fasudil (Fig. 1k). Moreover, phosphorylation of YAP was increased in the BAPN + Fasudil group compared with the BAPN group, indicating that inhibition of RhoA/ROCK1/YAP could further promote AD formation (Supplementary Fig. S5a). Histochemical analysis revealed that in contrast to the intact aortic wall in the vehicle and Fasudil groups, elastic fibers were disrupted and a dissection formed in the aortic wall of BAPN and BAPN + Fasudil groups (Fig. 1l; Supplementary Fig. S5b). Collagen deposition showed a slight reduction in the BAPN + Fasudil group compared to the vehicle group (Supplementary Fig. S5c). The pYAP/YAP ratio was significantly increased in the Fasudil and BAPN + Fasudil groups (Supplementary Fig. S5d). Additionally, the impact of aging and blood pressure on the RhoA/ROCK1/YAP signaling pathway was also explored in the aortas of other AD mouse models. In aging mice, only increased phosphorylation of YAP was found in the aging aortas, but no significant variation was found concerning RhoA or ROCK1 (Supplementary Fig. S5e and f). Similarly, increased phosphorylation of YAP, except for the difference in RhoA and ROCK1, was detected in mice treated with BAPN and angiotensin II (Ang II) compared to the control group (Supplementary Fig. S5g–i). Therefore, our animal model is a dedicated model confirming that a reduction in RhoA/ROCK1/YAP/F-actin signaling is one underlying mechanism of AD. This animal model also provides an available AD model for therapeutic research.

The mouse aortas were then harvested and analyzed by RNA-seq. Compared to the vehicle group, the mRNA levels of multiple matrix metalloproteinases (MMPs) were elevated in the BAPN- and BAPN + Fasudil-induced AD aortas, suggesting accelerated ECM degradation (Supplementary Fig. S5j). GO and KEGG analyses showed that DEGs between the BAPN + Fasudil group and the vehicle group were mostly related to ECM organization, cytoskeleton, and cell-ECM interaction (Fig. 1m; Supplementary Fig. S5k). Terms including cAMP response element binding, actin filament organization, ECM organization, and collagen formation were all downregulated in the BAPN + Fasudil group compared to the vehicle group (Supplementary Fig. S6a–c). In contrast, DEGs between the BAPN and vehicle groups were mainly related to AoSMC metabolism and ECM organization and structure, since BAPN mainly affected the activity of lysyl oxidase and ECM fiber maturation (Supplementary Fig. S6d and e). When DEGs were analyzed between the BAPN and BAPN + Fasudil groups, we found that fibers of cell force generation, cytoskeleton structure, and function were significantly different, reflecting the impact of Fasudil on AoSMC mechanics (Supplementary Fig. S6f and g). Therefore, the AD animal model induced by BAPN was mainly caused by AoSMC metabolic dysfunction, and downregulation of RhoA/ROCK1/YAP/F-actin signaling with decreased AoSMC stiffness could result in increased elastin fragmentation, reduced collagen production, and dysfunction of the cell-ECM unit, thereby facilitating AD formation. The results reflected the important role of the RhoA/ROCK1/YAP/F-actin signaling pathway and intrinsic AoSMC stiffness in preventing AD formation.

Deficiency of Rho GTPase and decreased YAP are reportedly related to aortopathology including AD. In this study, we directly measured downregulated RhoA/ROCK1/YAP/F-actin in primary AoSMCs derived from human AD aortas. Meanwhile, the downregulated signaling pathway led to decreased intrinsic AoSMC stiffness within AD aortas. Animal experiments revealed that mice co-administered with BAPN and Fasudil, a ROCK1 inhibitor, experienced a higher mortality rate due to AD. Notably, their aortas exhibited increased elastin fragmentation, reduced collagen deposition, and comprised cell-ECM unit function. Therefore, downregulation of RhoA/ROCK1/YAP/F-actin signaling leads to decreased AoSMC stiffness and promotes AD formation.

Vascular SMC mechanics are important for modulating vascular tone and maintaining vascular function. While numerous AD-predisposing genetic mutations have been associated with AoSMC mechanics and force generation, intrinsic AoSMC stiffness has not been directly measured in AD-affected aortas. Variations in AoSMC mechanics have attracted considerable attention in various pathological and physical conditions of the aorta, such as aging, hypertension, and atherosclerosis. Intrinsic AoSMC stiffness can adaptively increase in aortas with aging or hypertension to prevent aortic diseases [2, 3]. We observed diminished cell stiffness in AoSMCs derived from ruptured aortas with AD, accompanied by impaired cell–ECM interaction, ECM degeneration, and collapse of aortic integrity. The cytoskeleton is a major contributor to intrinsic cell stiffness, and impaired F-actin polymerization was observed to be concomitant with reduced intrinsic AoSMC stiffness. Moreover, upregulated expression of MMPs and impaired cell-ECM interaction were found according to RNA-seq analysis when the AoSMC stiffness was decreased, indicating the dysfunction of the elastin–contractile unit and ECM remodeling. Consistently, disrupted cytoskeletons in AoSMCs have also been reported to interfere with cell-matrix adhesions, leading to impaired cell mechanosensing and matrix remodeling. Additionally, both impaired cell–matrix adhesions and dysfunction of matrix fibers can further dysregulate AoSMC cytoskeleton and mechanics, which results in abnormal development of aortic arch and outflow tract as well as aortic diseases [11, 12]. Therefore, intrinsic AoSMC stiffness with an intact cytoskeleton is critical to maintaining the cell–matrix interaction and matrix formation.

The ROCK pathway and its effector YAP are pivotal biomechanical mediators. Our study primarily focuses on their regulation in intrinsic AoSMC stiffness, the dynamic balance of which is essential for orchestrating cell–matrix interactions and matrix remodeling. We found that downregulation of the RhoA/ROCK1/YAP/F-actin signaling pathway reduced intrinsic AoSMC stiffness, thereby promoting AD formation. This finding aligns with other studies emphasizing the protective role of ROCK and the adaptive elevation of YAP in the prevention of aortic diseases [4, 8]. In addition, no patients were diagnosed with hereditary AD in our study, excluding the causation of genetic mutations to the abnormality of the RhoA/ROCK1/YAP/F-actin axis. Several molecular mechanisms, e.g., the binding of Ang II, endothelin-1, fibroblast growth factor, and transforming growth factor *β* to their receptors on the cell membrane, have been reported to regulate ROCK signaling in the cardiovascular system. Therefore, we propose that the reduction in the RhoA/ROCK1/YAP/F-actin axis might arise from multiple upstream signaling abnormalities predisposing patients to AD. Hence, the RhoA/ROCK1/YAP/F-actin signaling pathway is a promising target for regulating intrinsic AoSMC stiffness in the cardiovascular system. Moreover, it is important to note that ROCK and YAP interact extensively with other signaling pathways in the cardiovascular system, such as Ca^2+^ sensitization, reactive oxygen species (ROS) production, and MMP activation [13, 14], all of which contribute to AD development. Therefore, any manipulation of the RhoA/ROCK1/YAP/F-actin signaling pathway should consider its potential impacts on other molecular mechanisms. Future studies should investigate the timing and duration of such manipulations. Additionally, it is noteworthy that many cardiovascular medications, such as calcium channel blockers and statins, have indirect effects on ROCK [15]. Thus, it is imperative to investigate the dosage and timing of certain cardiovascular medications to prevent any interference with AoSMC stiffness via RhoA/ROCK1/YAP/F-actin signaling in the future.

In summary, our study unveils a downregulated RhoA/ROCK1/YAP/F-actin cascade with decreased intrinsic AoSMC stiffness in AD aortas, which can lead to increased elastin fragmentation and deficient collagen production, promoting AD formation. This research underscores the critical role of AoSMC stiffness in maintaining aortic integrity and highlights potential targets to prevent AD occurrence. Moreover, the alterations in intrinsic AoSMC stiffness during AD formation also hint that the impact of cardiovascular medicine on AoSMC stiffness should be considered to prevent further collapse of aortic integrity.

## Supplementary Material

loae022_suppl_Supplementary_Material

## Data Availability

The online version of this article contains supplementary material, which is available to authorized users.

## References

[1] Karimi A, Milewicz DM. Structure of the elastin-contractile units in the thoracic aorta and how genes that cause thoracic aortic aneurysms and dissections disrupt this structure. Can J Cardiol 2016;32:26–34. 26724508 10.1016/j.cjca.2015.11.004 PMC4839280

[2] Sehgel NL, Sun Z, Hong Z et al. Augmented vascular smooth muscle cell stiffness and adhesion when hypertension is superimposed on aging. Hypertension 2015;65:370–7. 25452471 10.1161/HYPERTENSIONAHA.114.04456 PMC4289111

[3] Sehgel NL, Zhu Y, Sun Z et al. Increased vascular smooth muscle cell stiffness: a novel mechanism for aortic stiffness in hypertension. Am J Physiol Heart Circ Physiol 2013;305:H1281–7. 23709594 10.1152/ajpheart.00232.2013 PMC3840243

[4] Zhang C, Li Y, Chakraborty A et al. Aortic stress activates an adaptive program in thoracic aortic smooth muscle cells that maintains aortic strength and protects against aneurysms and dissection in mice. Arterioscler Thromb Vasc Biol 2023;43:234–52. 36579645 10.1161/ATVBAHA.122.318135 PMC9877188

[5] Zhu Y, Qiu H, Trzeciakowski JP et al. Temporal analysis of vascular smooth muscle cell elasticity and adhesion reveals oscillation waveforms that differ with aging. Aging Cell 2012;11:741–50. 22639979 10.1111/j.1474-9726.2012.00840.x PMC3444643

[6] Sanyour HJ, Li N, Rickel AP et al. Membrane cholesterol and substrate stiffness co-ordinate to induce the remodelling of the cytoskeleton and the alteration in the biomechanics of vascular smooth muscle cells. Cardiovasc Res 2019;115:1369–80. 30395154 10.1093/cvr/cvy276 PMC11268160

[7] Nogi M, Satoh K, Sunamura S et al. Small GTP-binding protein GDP dissociation stimulator prevents thoracic aortic aneurysm formation and rupture by phenotypic preservation of aortic smooth muscle cells. Circulation 2018;138:2413–33. 29921611 10.1161/CIRCULATIONAHA.118.035648

[8] Pan L, Bai P, Weng X et al. Legumain is an endogenous modulator of integrin αvβ3; triggering vascular degeneration, dissection, and rupture. Circulation 2022;145:659–74. 35100526 10.1161/CIRCULATIONAHA.121.056640

[9] Jiang WJ, Ren WH, Liu XJ et al. Disruption of mechanical stress in extracellular matrix is related to Stanford type A aortic dissection through down-regulation of Yes-associated protein. Aging (Albany NY). 2016;8:1923–39. 27608489 10.18632/aging.101033 PMC5076445

[10] Perez-Gonzalez NA, Rochman ND, Yao K et al. YAP and TAZ regulate cell volume. J Cell Biol 2019;218:3472–88. 31481532 10.1083/jcb.201902067 PMC6781432

[11] Massett MP, Bywaters BC, Gibbs HC et al. Loss of smooth muscle α-actin effects on mechanosensing and cell-matrix adhesions. Exp Biol Med (Maywood) 2020;245:374–84. 32064918 10.1177/1535370220903012 PMC7370591

[12] Arnold TD, Zang K, Vallejo-Illarramendi A. Deletion of integrin-linked kinase from neural crest cells in mice results in aortic aneurysms and embryonic lethality. Dis Model Mech. 2013;6:1205–12. 23744273 10.1242/dmm.011866 PMC3759340

[13] Uehata M, Ishizaki T, Satoh H et al. Calcium sensitization of smooth muscle mediated by a Rho-associated protein kinase in hypertension. Nature 1997;389:990–4. 9353125 10.1038/40187

[14] Shimokawa H, Satoh K. 2015 *ATVB* Plenary Lecture: translational research on Rho-kinase in cardiovascular medicine. Arterioscler Thromb Vasc Biol 2015;35:1756–69. 26069233 10.1161/ATVBAHA.115.305353

[15] Shimokawa H, Takeshita A. Rho-kinase is an important therapeutic target in cardiovascular medicine. Arterioscler Thromb Vasc Biol 2005;25:1767–75. 16002741 10.1161/01.ATV.0000176193.83629.c8

