# Supplementary material for: Downregulation of RhoA/ROCK1/YAP/F-actin causing decreased aortic smooth muscle cell stiffness promotes aortic dissection formation
Source: Life Metab. 2024 Jun 3;3(5):loae022. doi: 10.1093/lifemeta/loae022 (PMC11749839; doi:10.1093/lifemeta/loae022)
Supplement: loae022_suppl_Supplementary_Material [file loae022_suppl_Supplementary_Material.zip › loae022_suppl_Supplementary_material/supplementary_information - tu.pdf]

Supplementary Materials for

Downregulation of RhoA/ROCK1/YAP/F-actin causing decreased aortic smooth muscle cell stiffness promotes aortic dissection formation

Methods and materials

Animal models and treatments

Three-week-old specific-pathogen-free (SPF) male C57BL/6J mice were purchased from Shanghai SLAC Laboratory Animal Co., Ltd. The mice were maintained in an SPF facility with a 12 h light/12 h dark cycle for one week to acclimate to the environment and then four weeks throughout the experiments. All mice were randomly assigned to the following groups: (1) Vehicle group (*n* = 11), mice were fed distilled water only; (2) Fasudil group (*n* = 10), mice were fed Fasudil dissolved in water at a dose of 150 mg/kg body weight/day; (3) β-aminopropionitrile (BAPN) group (*n* = 12), mice were fed BAPN dissolved in distilled water at a concentration of 0.25% (w/v); and (4) BAPN + Fasudil group, mice were first fed BAPN (0.25% in distilled water) for one week and then fed BAPN (0.25% in distilled water) and Fasudil (150 mg/kg body weight/day) together to induce AD （Supplymentary table S1）. After four weeks of treatment, aortas were collected from all surviving mice to assess the AD formation and for further analysis. All procedures conformed to the ARRIVE Guidelines for the Care and Use of Laboratory Animals and were approved by the Institutional Animal Care and Use Committee of Zhongshan Hospital, Fudan University.

Primary AoSMC isolation and culture

Thoracic aorta tissue was obtained from Stanford type A AD patients and organ donors for heart transplantation who underwent surgery at Zhongshan Hospital, Fudan University. The human experiments were approved by the Institutional Research Ethics Committee Review Board (B2018-040R). The baseline information of the patients and donors is summarized and compared in Table 1. The aortic smooth muscle cells (AoSMCs)were isolated from lesions of AD aortas and corresponding segments of normal aortas. Briefly, the aortic intima and adventitia were stripped using forceps with media left in Smooth Muscle Cell Medium (SMCM) at 37°C. After washing with phosphate-buffered saline (PBS) three times, the aortic media was cut into 1 mm × 1 mm pieces. The pieces were placed in a 10-cm dish at approximately 1 cm interval. The dish was then inverted and incubated at 37°C, 5% CO_2, and

95% relative humidity for 4 h. Following the incubation, SMCM was slowly added to the dish without disturbing the attached tissue pieces. Then the tissue pieces were cultured at 37°C, 5% CO_2, and 95% relative humidity. The culture medium was replaced 96 h later and every 72 h thereafter. Primary AoSMCs migrated from the edge of tissue pieces on approximately the fourth day. Primary AoSMCs were passaged for further experiments when cells migrating from different pieces of tissue merged. All experiments were performed before passage 6, and the phenotype of AoSMCs was confirmed by α-SMA expression.

Atomic force microscopy (AFM)

Dimension Icon & FastScan Bio (Bruker, USA) was used to measure cellular stiffness. A 5-μm silica bead was attached to a backside gold-coated silicon nitride tipless cantilever (MLCT-O, Bruker) to prevent damage to cells during the measurement. The spring constant (0.1 N/m) of the probe was calibrated using the thermal noise method (JPK Instruments AG) before scanning each sample. The laser detector was centralized, and the laser reflection sum was adjusted to the maximum value. Cells cultured in 60-mm dishes were evaluated in a liquid environment at 37°C. Primary human AoSMCs of the second passage were measured. The force mapping procedures for all the samples were identical. Ten testing points were randomly selected and measured for each sample.

Calculation of intrinsic AoSMC stiffness

The following parameters were set for the AFM procedures: a tip speed of 0.5 s per curve, peak force of 800 pN, Z-length of 15.0 μm, and curve recording of 30 μm/s, and the force-distance curve was recorded with a ramp rate of 2 kHz. The absolute value of Young's modulus was finally determined using the Hertz model:

$$F=\frac{4}{3}\frac{E}{(1-v^2)}\sqrt{R}\delta^{3/2},$$

where F is the measured force, R is the radius of the bead, ν is Poisson's ratio (0.5 for incompressible materials), and δ is the indentation depth. Data analyses were carried out using NanoScope Analysis 1.8 (Bruker, USA).

Blood pressure measurement and electrocardiography

Blood pressure was measured at the beginning and end points of the experiment using CODA noninvasive blood pressure acquisition system (Kent Scientific Corporation, USA). Briefly, mice were acclimated to a quiet environment for 1 h before experiment. Then the mice were encouraged into retrained tubes with end holders to prevent excess movement. The occlusion cuff was placed at the root of tail with adjacent VPR sensor cuff. The heating pads were warmed from 5 min before the experiment to the measurement end. The occlusion cuff was inflated to 250 mmHg and deflated over 20 s. The VPR sensor cuff measured changes in tail volume during deflation. The minimum volume change was set as 15 μL. Each recording set contained 15 to 25 inflation and deflation cycles, of which the first 5 cycles were regarded as "accustomed" cycles and discarded. The morphology and diameter of thoracic aorta were assessed using echocardiography (VINNO 6 VET, VINNO, Suzhou, China). The mice were anesthetized with isoflurane and the chest and abdominal fur were cleared. B-mode ultrasonography was employed to visualize the morphology and measure the diameter of descending aorta.

Generation of knockout (KO) cells using CRISPR/Cas9

Single guide RNAs were cloned and inserted into pSpCas9(BB)-2A-GFP(PX458); Addgene plasmid #48138. The guide sequence for *YAP* was 5′-CATCAGATCGTGCACGTCCG-3′. Primary AoSMCs were transfected with the resulting vector containing sgRNA sequences using Lipofectamine 2000 Transfection Reagent. The medium was changed after 24 hours of treatment. Single GFP-expressing cells were selected via fluorescence-activated cell sorting and seeded into a 96-well plate. Single-cell colonies were expanded and then screened by western blotting. The

phenotype of AoSMCs with *YAP* depletion was confirmed by α-SMA expression (Supplemental Fig. S1e).

Immunofluorescence

Cells seeded on glass slides were fixed with 4% paraformaldehyde in PBS for 10 min and permeabilized in 0.1% Triton-X 100 in PBS for 15 min. The cells were blocked with 5% BSA in PBS for 1 h and incubated with primary antibodies overnight at 4°C. After thorough washing with PBS three times, the samples were incubated with the corresponding Alexa fluorochrome-conjugated secondary antibodies for 1 h at room temperature. F-actin was stained with FITC (excitation at 516 nm)-conjugated phalloidin actin-tracker green. Complete F-actin polymerization was demonstrated as continuous filaments. Nuclei were stained with 4',6'-diamidino-2-phenylindole for 8 min. The slides were mounted with ProLong™ Glass Antifade Mountant and visualized using the EVOS™ M5000 Imaging System (Thermo Fisher Scientific). Z-stacks were automatically optimized, with the maximum intensity algorithm being applied. The rate of YAP nuclear localization was calculated using the following formula: formula:

$$\frac{\sum_{nuc}^{I}/A_{nuc}}{\sum_{cell}^{I}/A_{cell}}$$

in which $\sum_{nuc}^{I}\ and\ \sum_{cell}^{I}$ represents the sum intensity value for the pixels in the nucleus and the whole-cell regions. Anuc and Acell represent the area of the nucleus and the whole area of cells, respectively. ImageJ (National Institutes of Health, USA) was used for signal analysis and channel merging.

Western blotting

Tissue or cells were lysed using lysis buffer (50 mmol/L Tris pH 6.8, 2% SDS, 5% glycerol, 1% 2-ME 4°C, 5 mmol/L EDTA pH 8.0, protease inhibitor cocktail and halt phosphatase inhibitor were added prior to use) for 15 min at 4°C. Protein concentrations were quantified with a BCA protein assay kit (Beyotime) according to the manufacturer's instructions. Thirty micrograms of each protein sample was used for SDS-PAGE electrophoresis. The proteins

were transferred onto nitrocellulose membranes (Solarbio) using the Trans-Blot system (Bio-Rad). The membranes were blocked with 5% skim milk in PBST for 1 h at room temperature. Then, the membranes were incubated with primary antibodies overnight at 4°C and HRP-conjugated secondary antibodies for 1 h at room temperature. Bands were detected by chemiluminescence ImageQuantTM LAS 4000 (GE, USA). Imaging and quantification of bands were performed using Image LabTM Software (Bio-Rad).

RNA sequencing

Total RNA was extracted using the TRIzol Plus RNA Purification Kit (Thermo Fisher Scientific) following the manufacturer's protocol. SBS50 sequencing was performed at DNBSEQ using illumine Hiseq^{TM} 2000. Reads that contained the sequence of adaptor, high content of unknown bases, and low-quality reads were filtered and stored in FASTQ format. RNA-seq reads were aligned to mouse GRCm39_release-103 with Bowtie2 and then raw reads were obtained with RSEM. The FPKM method was used to calculate gene expression for direct comparison among samples. Finally, the DESeq2 method was used to screen differentially expressed genes (DEGs) between two groups according to the following default criteria: fold change ≥ 2 and Q value (adjusted P value) ≤ 0.05. Gene Ontology (GO) enrichment analysis and pathway enrichment analysis (KEGG based) were performed for DEGs.

Histochemical analysis

The aortas were fixed overnight in 4% paraformaldehyde and then dehydrated and embedded in paraffin. Paraffin-embedded sections were cut serially at 4-μm intervals. Masson's trichrome and Verhoeff's Van Gieson staining were performed using commercial staining kits (Solarbio) according to the manufacturer's instructions. For immunohistochemical staining, sections were deparaffinized and dehydrated, and heated in Tris-EDTA buffer (pH 9.0) for antigen retrieval. The slides were washed with TBS plus 0.1% Triton X-100 (TBST) three times and blocked in 5% BSA in TBS for 1 h at room temperature. The slides

were incubated with the primary antibody overnight at 4°C and then HRP-conjugated secondary antibody for 1 h at room temperature. After washing with TBST three times, the slides were incubated with DAB (Solarbio) for 10 min at room temperature and washed with running tap water for 5 min. Then the slides were dehydrated and mounted.

Statistical analysis

Categorical data are presented using absolute numbers, and quantitative data are presented using means ± standard deviations (SDs). A P value < 0.05 was set as the threshold for statistical significance. Baseline data were compared between the normal and AD groups using two-tailed Student's t test or the chi-squared test. The survival rate of the animal model was estimated using the Mantel–Cox test. All statistical analyses were conducted using GraphPad Prism version 8.0.

Chemicals and antibodies

Product	Company, CAT
Smooth Muscle Cell Medium	ScienCell Research Laboratories, 1101
β-aminopropionitrile (BAPN)	Sigma-Aldrich, A3134
ProLong™ Glass Antifade Mountant	Thermo Fisher Scientific, P36984
Y-27632 2HCl	Selleck, S1049
Fasudil (HA-1077) HCI	Selleck, S1573
actin-Tracker Green	Beyotime, C1033
ROCK1 (C8F7) rabbit monoclonal antibody	Cell Signaling Technology, 4035
RhoA rabbit polyclonal antibody	Proteintech, 10749-1-AP
YAP1 rabbit polyclonal antibody	Proteintech, 13584-1-AP
Phospho-YAP (Ser127) (D9W2I) Rabbit mAb	Cell Signaling Technology, 4911
GAPDH mouse monoclonal antibody	Proteintech, 60004-1-Ig

α-Smooth Muscle Actin (D4K9N) XP® Rabbit mAb	Cell Signaling Technology, 19245
HRP and goat anti-mouse IgG (H+L) secondary antibody	Thermo Fisher Scientific, 31430
HRP and goat anti-rabbit IgG (H+L) secondary antibody	Thermo Fisher Scientific, 31460
Alexa Fluor 647 AffiniPure goat anti-rabbit IgG (H+L)	Jackson ImmunoResearch, 111-605-003
Fluorescein (FITC) AffiniPure goat anti-mouse IgG (H+L)	Jackson ImmunoResearch, 115-095-003

Supplementary Table S1 Follow-up of animal models.

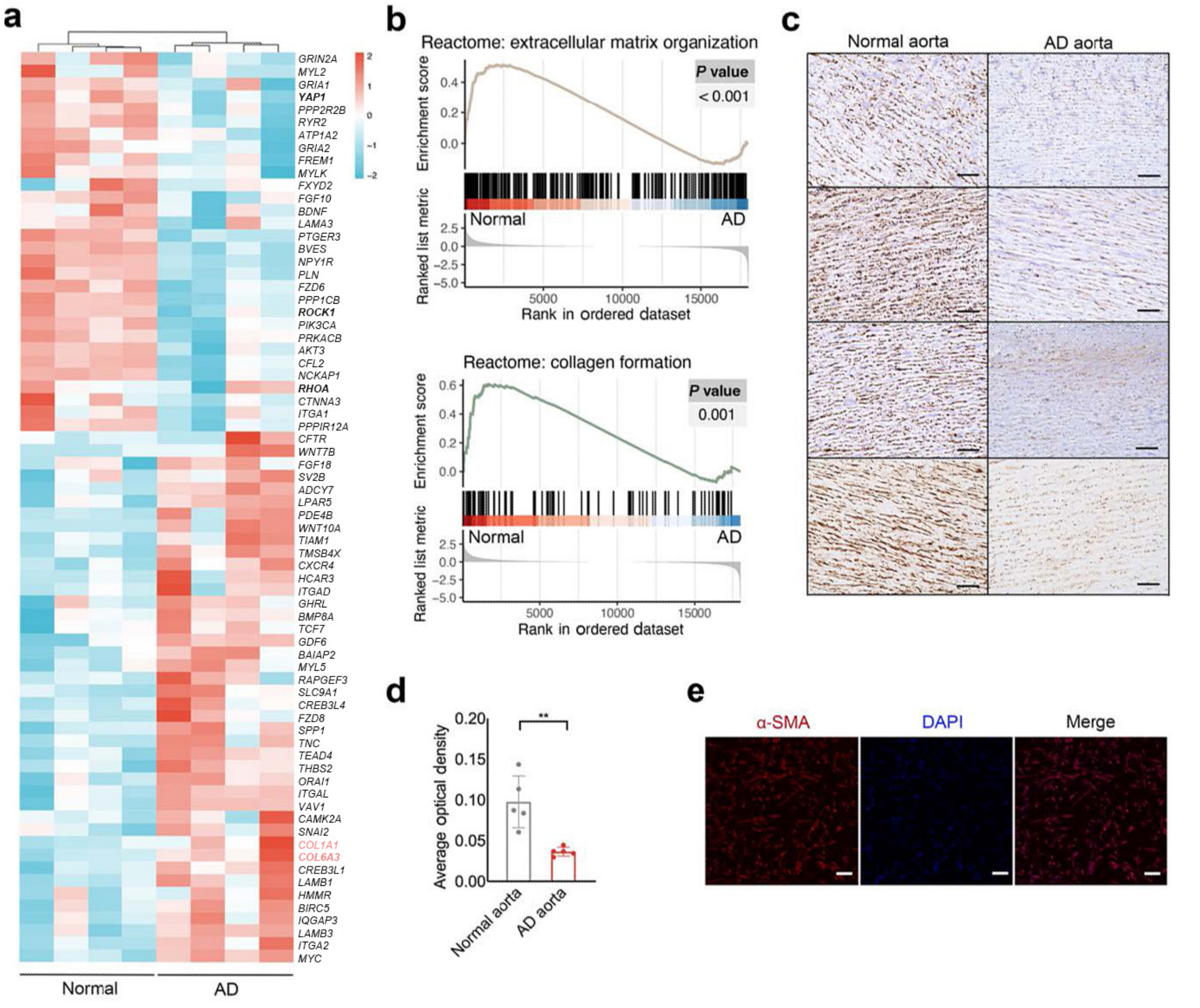

Supplementary Figure S1 (a) Heatmap of gene expression based on the AD GEO database (GSE147026). (b) GSEA showed downregulation in terms of extracellular matrix organization and collagen formation in AD aortas. (c) Immunohistochemical staining of YAP in normal and AD aortas. Scale bar = 200 μm. (d) Statistical analysis of average YAP expression in normal and AD aortas ($^{**}P < 0.01$). (e) Immunofluorescence staining of α-SMA for primary AoSMCs. Scale bar = 200 μm.

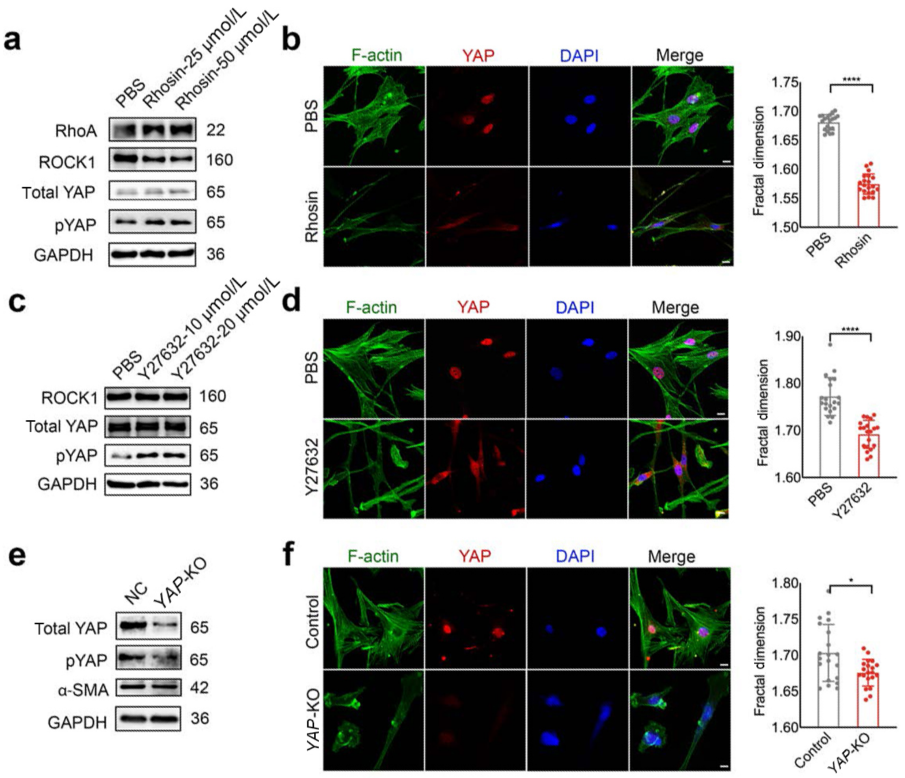

Supplementary Figure S2 (a) Western blotting of AoSMCs treated without and with Rhosin (25 μmol/L, 50 μmol/L, 48 h). (b) Immunofluorescence of AoSMCs treated without and with Rhosin (50 μmol/L, 48 h). Scale bar = 10 μm. Graph: The fractal dimension of AoSMCs treated without and with Rhosin ($^{****}P < 0.0001$). (c) Western blotting analysis of AoSMCs treated without and with Y27632 (10 μmol/L, 20 μmol/L, 48 h). (d) Immunofluorescence of AoSMCs treated without and with Y27632 (10 μmol/L, 48 h). Scale bar = 10 μm. Graph: The fractal dimension of AoSMCs treated without and with Y27632. ($^{****}P < 0.0001$). (e) Western blotting analysis of AoSMCs with *YAP* depletion and normal controls. (f) Immunofluorescence of normal AoSMCs and *YAP* knockout (*YAP*-KO) AoSMCs. Scale bar = 10 μm. Graph: The fractal dimension of control and *YAP*-KO AoSMCs ($^{*}P < 0.05$).

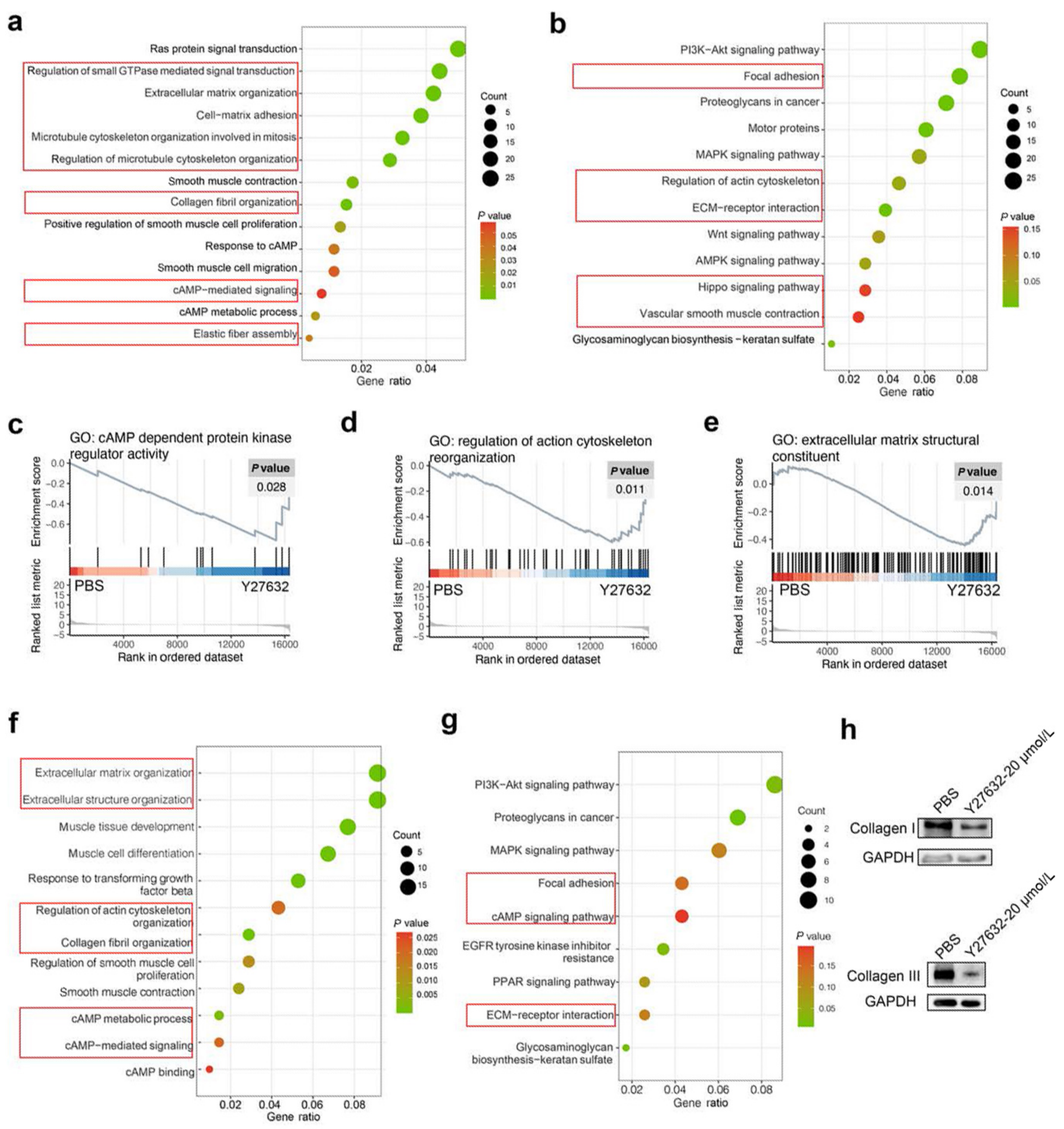

Supplementary Figure S3 (a) GO analysis of DEGs between primary AoSMCs treated with Y27632 and control AoSMCs. (b) KEGG analysis of DEGs between primary AoSMCs treated with Y27632 and control AoSMCs. (c−e) GSEA of (c) cAMP-dependent protein kinase regulation activity ($^*P < 0.05$), (d) regulation of actin cytoskeleton organization ($^*P < 0.05$), and (e) extracellular matrix structural constituent ($^*P < 0.05$) between primary AoSMCs treated with Y27632 and control AoSMCs. (f) GO analysis of downregulated DEGs between primary AoSMCs treated with Y27632 and control AoSMCs. (g) KEGG

analysis of downregulated DEGs between primary AoSMCs treated with Y27632 and control AoSMCs. (h) Western blot analysis of collagen I and collagen III in primary AoSMCs treated with Y27632 and control AoSMCs.

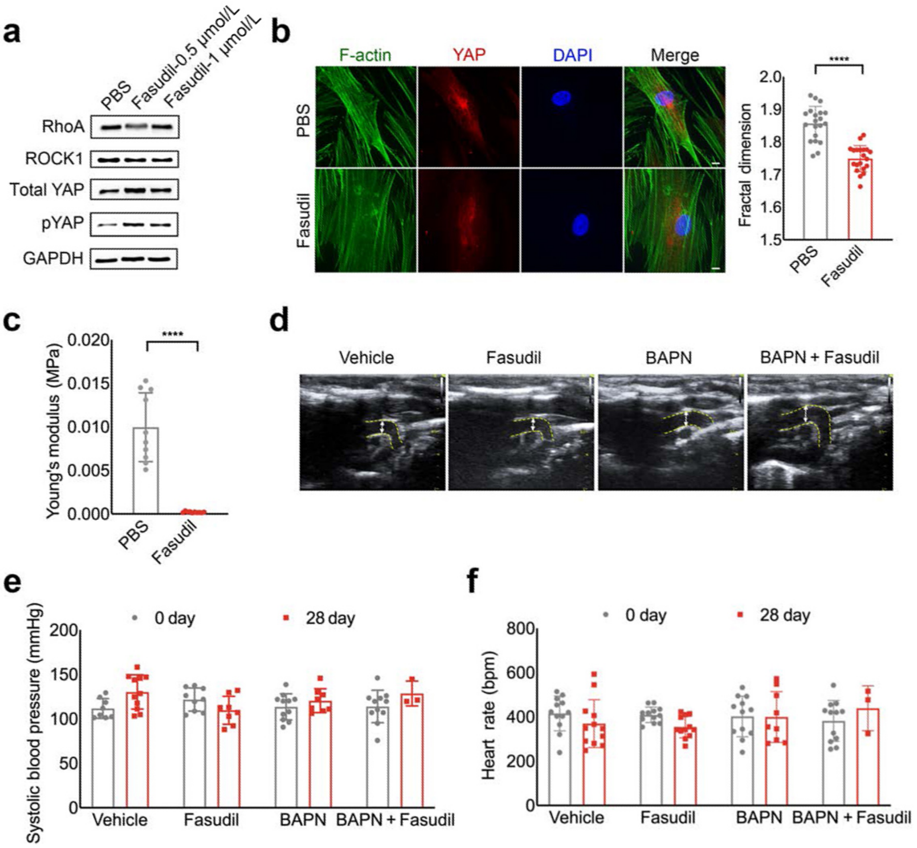

Supplementary Figure S 4. (a) Expression of RhoA, ROCK, YAP, and pYAP in primary AoSMCs treated with Fasudil (0.5 μmol/L, 1 μmol/L, 48 h). (b) Immunofluorescence of primary AoSMCs treated with Fasudil (1 μmol/L) and control. Scale bar = 5 μm. Graph: The fractal dimension of primary AoSMCs treated Fasudil and control ($^{****}P < 0.0001$). (c) Young's modulus of primary AoSMCs treated with Fasudil and control ($^{****}P < 0.0001$). (d) Echocardiography of aortas in animal models. (e) Blood pressure comparison at the beginning and end points of the experiment in each group. (f) Heart rate comparison at the beginning and end points of the experiment in each group.

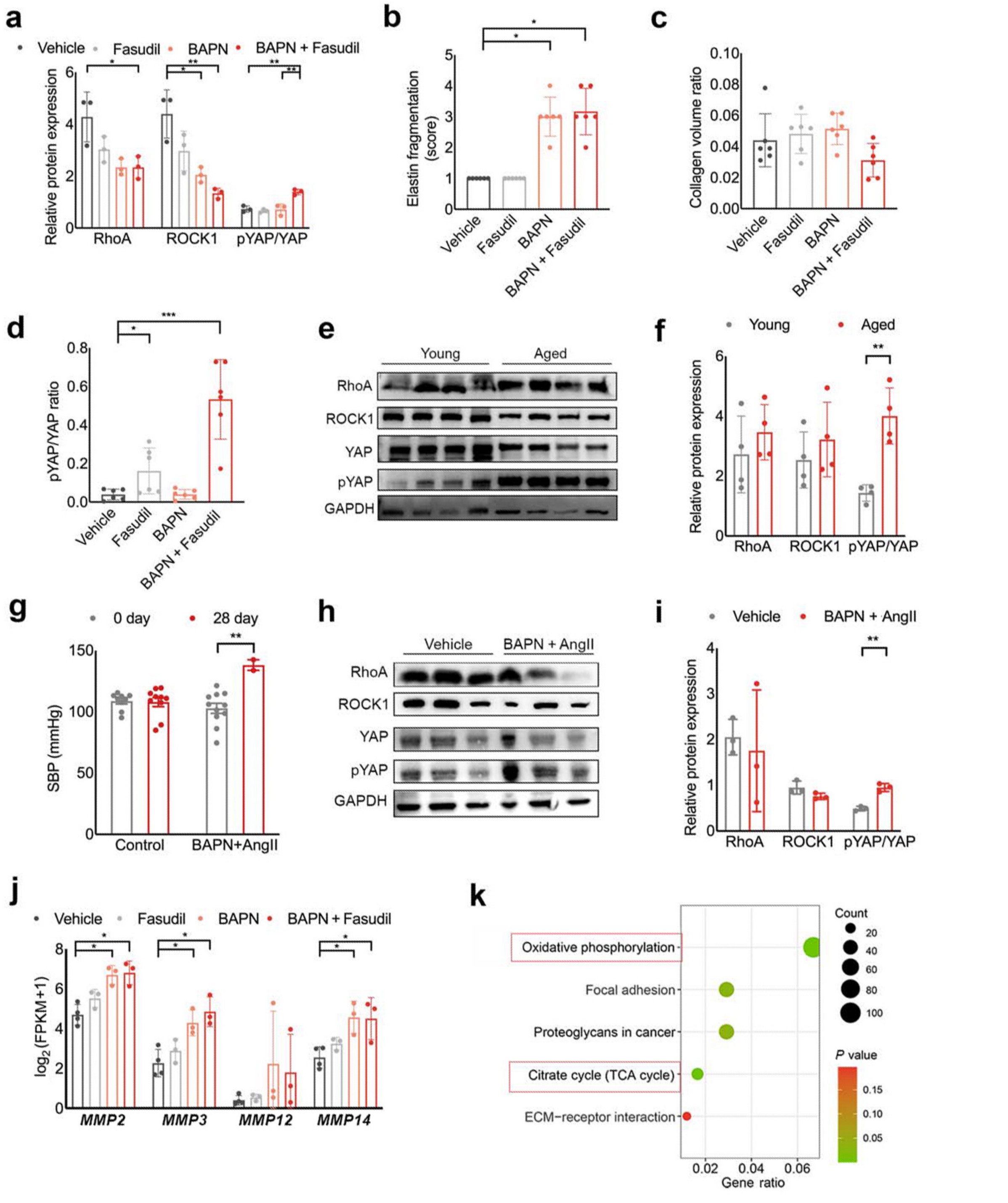

Supplementary Figure S5 (a) Statistical analysis of relative protein expression of RhoA and ROCK1, and pYAP/YAP ratio (RhoA: Vehicle vs. BAPN + Fasudil, $^{*}P < 0.05$; ROCK1: Vehicle vs. BAPN, $^{*}P < 0.05$; Vehicle vs. BAPN + Fasudil, $^{**}P < 0.01$; pYAP/YAP: Vehicle vs BAPN + Fasudil, $^{**}P < 0.01$; BAPN vs. BAPN + Fasudil, $^{**}P$ <0.01). (b) Graph: Statistical analysis of elastin fragmentation based on EVG (Vehicle vs. BAPN, $^{*}P < 0.05$; Vehicle vs.

BAPN + Fasudil, $^{*}P < 0.05$). (c) Graph: Statistical analysis of collagen deposition based on Masson's trichrome staining. (d) Graph: Statistical analysis of the pYAP/YAP ratio (Vehicle vs Fasudil, $^{*}P < 0.05$; Vehicle vs. BAPN + Fasudil, $^{***}P < 0.001$). (e) Western blotting analysis of RhoA/POCK1/YAP signaling in aortas harvested from young (4 weeks) and old (12 months) mice. (f) Statistical analysis of relative protein expression of RhoA and ROCK1, and pYAP/YAP ratio between young and old mice. (g) Blood pressure comparison at the beginning and end points of the experiment between the control and mice treated with BAPN and Ang II. (h) Western blotting analysis of RhoA/POCK1/YAP signaling in aortas harvested from mice treated with BAPN and AngII as well as the control. (i) Statistical analysis of relative protein expression of RhoA and ROCK1, and pYAP/YAP ratio between mice treated with BAPN and AngII as well as the control. (j) Expression of *MMP2*, *MMP3*, *MMP12*, and *MMP14* among different groups according to RNA-seq of AD animal models. (k) KEGG analysis of DEGs between the vehicle and BAPN + Fasudil groups.

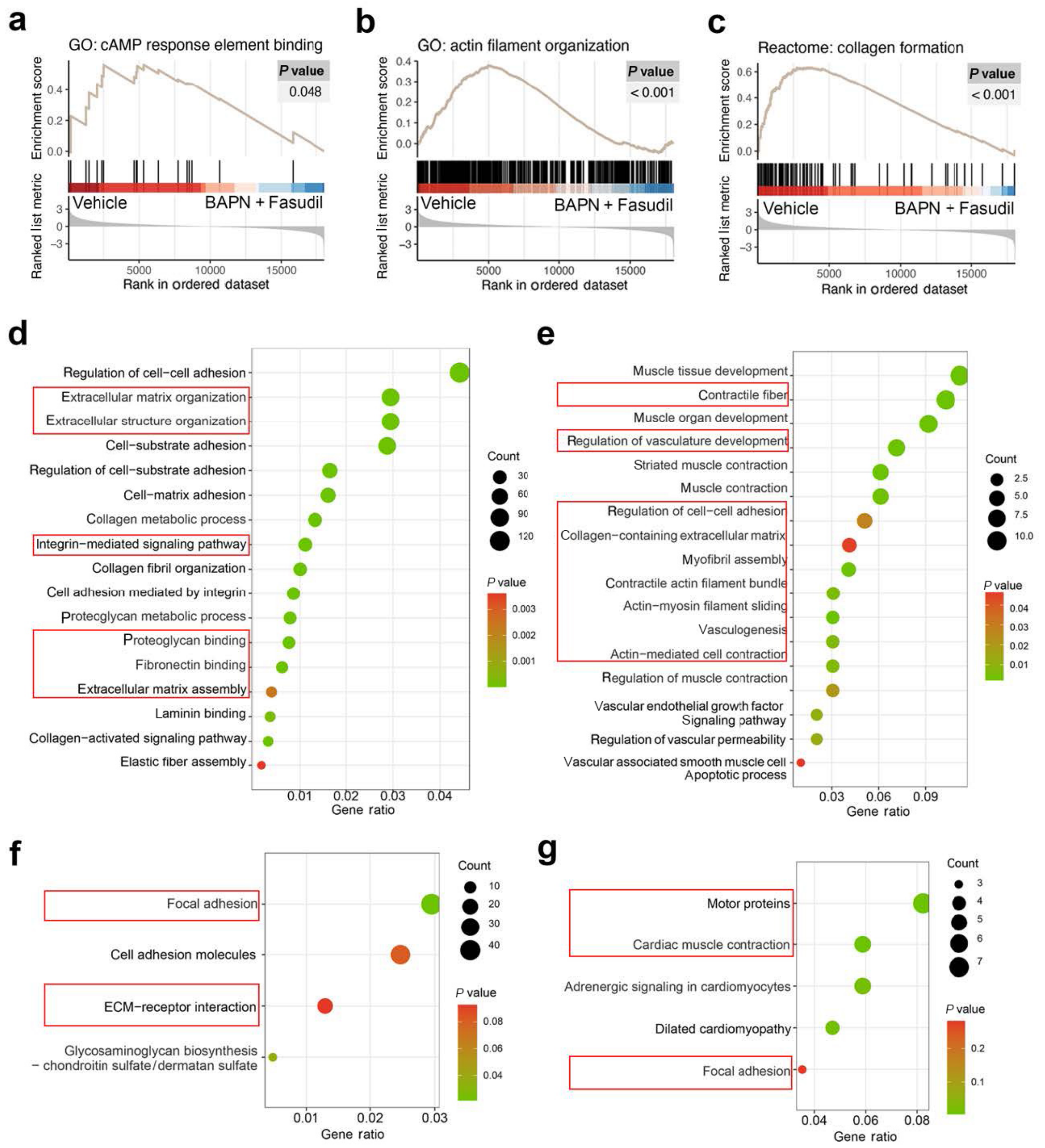

Supplementary Figure S6 GSEA of (a) cAMP response element binding ($^{*}P < 0.05$), (b) actin filament organization ($^{***}P < 0.001$), and (c) collagen formation ($^{***}P < 0.001$) between the vehicle and BAPN + Fasudil groups. (d) GO analysis of DEGs between the vehicle and BAPN groups. (e) KEGG analysis of DEGs between the vehicle and BAPN groups. (f) GO analysis of DEGs between the BAPN and BAPN + Fasudil groups. (g) KEGG analysis of DEGs between the BAPN and BAPN + Fasudil groups.
